# Is bowel preparation necessary for early colonoscopy in patients with suspected colonic diverticular bleeding?: A multicenter retrospective study with propensity score matching analysis

**DOI:** 10.1002/deo2.311

**Published:** 2023-11-02

**Authors:** Takahiro Gonai, Yosuke Toya, Norihiko Kudara, Keinosuke Abe, Sera Sawaguchi, Takao Fujiwara, Makoto Eizuka, Minami Hirai, Manami Miura, Jun Urushikubo, Shun Yamada, Tomo Kumei, Satoko Yamaguchi, Kyohei Sugai, Kensuke Asakura, Shunsuke Orikasa, Takayuki Matsumoto

**Affiliations:** ^1^ Division of Gastroenterology and Hepatology Department of Internal Medicine Iwate Medical University School of Medicine Iwate Japan; ^2^ Department of Gastroenterology Iwate Prefectural Kuji Hospital Iwate Japan; ^3^ Department of Gastroenterology Iwate Prefectural Ofunato Hospital Iwate Japan; ^4^ Department of Gastroenterology Iwate Prefectural Miyako Hospital Iwate Japan; ^5^ Department of Gastroenterology Morioka Red Cross Hospital Iwate Japan; ^6^ Department of Gastroenterology Hachinohe Red Cross Hospital Aomori Japan; ^7^ Department of Gastroenterology Iwate Prefectural Ninohe Hospital Iwate Japan; ^8^ Department of Gastroenterology Noshiro Kosei Medical Center Akita Japan; ^9^ Department of Gastroenterology Kazuno Kosei Hospital Akita Japan; ^10^ Department of Gastroenterology Kitakami Saiseikai Hospital Iwate Japan

**Keywords:** acute lower gastrointestinal bleeding, colonic diverticular bleeding, colonic diverticular hemorrhage, diverticular disease, early colonoscopy

## Abstract

**Objectives:**

There are few reports on bowel preparation for early colonoscopy in patients with suspected colonic diverticular bleeding (CDB). We aim to clarify in a retrospective, multicenter study.

**Methods:**

In a multicenter retrospective cohort study at 10 institutions, we analyzed clinical features of patients diagnosed with CDB, who underwent early colonoscopy within 24 h. We compared patients who were prepared with polyethylene glycol lavage (PEL) and those without PEL. We evaluated the effects of PEL for early colonoscopy in patients with suspected CDB.

**Results:**

A total of 129 (53%) underwent under preparation with PEL and 113 patients without PEL. The PEL group was younger, had fewer comorbidities, and had better performance status. After adjusting for these variables with propensity score matching, the PEL group had a significantly shorter hospital stay (7.9 ± 4.7 vs. 10.1 ± 5.2 days; *p* = 0.001), and a higher cecal intubation rate (91.1% vs. 50.0%; *p* < 0.001). There were no significant differences in adverse event rates, identification of stigmata of recent hemorrhage, or frequency in endoscopic hemostatic treatment.

**Conclusions:**

PEL may be preferred for early colonoscopy in patients suspected of having CDB.

## INTRODUCTION

Colonic diverticular bleeding (CDB) is a common cause of acute lower gastrointestinal bleeding (ALGIB). In a recent Japanese multicenter retrospective cohort study, 63.6% of patients with ALGIB were ultimately diagnosed with CDB.^1^ With an aging society, the reported prevalence of colonic diverticula is increasing,^2^ and CDB will be recognized as a more common cause of ALGIB.

Patients with CDB manifest more severe anemia than those of other entities.^3^, ^4^ Inadequate initial treatment may lead to the worsening of the patient's clinical status and to a delay in discharge from the hospital. Although endoscopic diagnosis and intervention are important in the care in the acute phase of CDB, the preferred endoscopic approach to this condition has not been well established. Recent studies have shown that urgent colonoscopy does not always improve clinical outcomes compared with elective colonoscopy.^5^, ^6^, ^7^, ^8^

Although endoscopic intervention^9^, ^10^, ^11^ and management of antithrombotic agents^12^ in patients with CDB have been discussed, there have been few data of the contribution of bowel preparation to the diagnostic value of urgent colonoscopy in patients with CDB. We therefore aim to clarify the benefits and safety of bowel preparation in patients with CDB, who underwent early colonoscopy.

## METHODS

### Study design

This multicenter, retrospective cohort study took place at 10 institutions in the prefectures of Iwate, Akita, and Aomori in northern Japan. We reviewed the medical records and endoscopic reports of patients hospitalized with CDB in these institutions between January 2015 and August 2019. Among 326 patients registered in the database, we included patients who underwent colonoscopy within 24 h after admission for the present study. We evaluated whether bowel preparation for early colonoscopy in patients with suspected CDB affected length of hospital stay, blood transfusion requirements, rebleeding rates, endoscopic findings, and adverse events.

Informed consent for study participation was obtained from participants through an online opt‐out system. The study protocol was approved by the ethical committee of Iwate Medical University (MH2019‐135) and was conducted according to the principles of the Declaration of Helsinki.

### Definitions

We defined CDB according to the guidelines of the Japan Gastroenterology Association^13^ as sudden, painless, visible hematochezia with either active bleeding from colonic diverticula observed by colonoscopy or colonoscopic confirmation of colonic diverticula and exclusion of other lower intestinal bleeding conditions. Early colonoscopy was defined as colonoscopy performed within 24 h of admission. Rebleeding was defined as visible hematochezia occurring at least 24 h after the confirmation of clinical hemostasis. Bowel preparation consisted of oral consumption of 1–2 L of electrolyte lavage solution. The enema was not regarded as a bowel preparation in this study. The observation time included the time for endoscopic hemostasis. Adverse events were defined as abdominal fullness, vomiting, and vagal reflexes due to bowel preparation. Stigmata of recent hemorrhage (SRH) on endoscopy were defined as active bleeding, adherent clots, or visible vessels.^14^

### Statistical analysis

We used propensity score matching to reduce selection bias during the statistical comparison of clinical outcomes between patients who underwent polyethylene glycol lavage (PEL group) and those who did not (non‐PEL group). We matched the variables at hospital admission and before endoscopy (i.e., age, sex, comorbidities, medications, performance status, and laboratory data). The propensity score matching was performed using a logistic regression model and a caliper width of 0.2. Comparisons among groups were performed using Pearson's chi‐squared test, the Mann–Whitney *U* test, or Student's *t*‐test. A standardized difference of less than 0.1 was denoted good balance between both groups. A *p*‐value of less than 0.05 was considered statistically significant. All statistical analyses were performed using JMP for Mac, version 13 (JMP Statistical Discovery LLC).

## RESULTS

**FIGURE 1:**
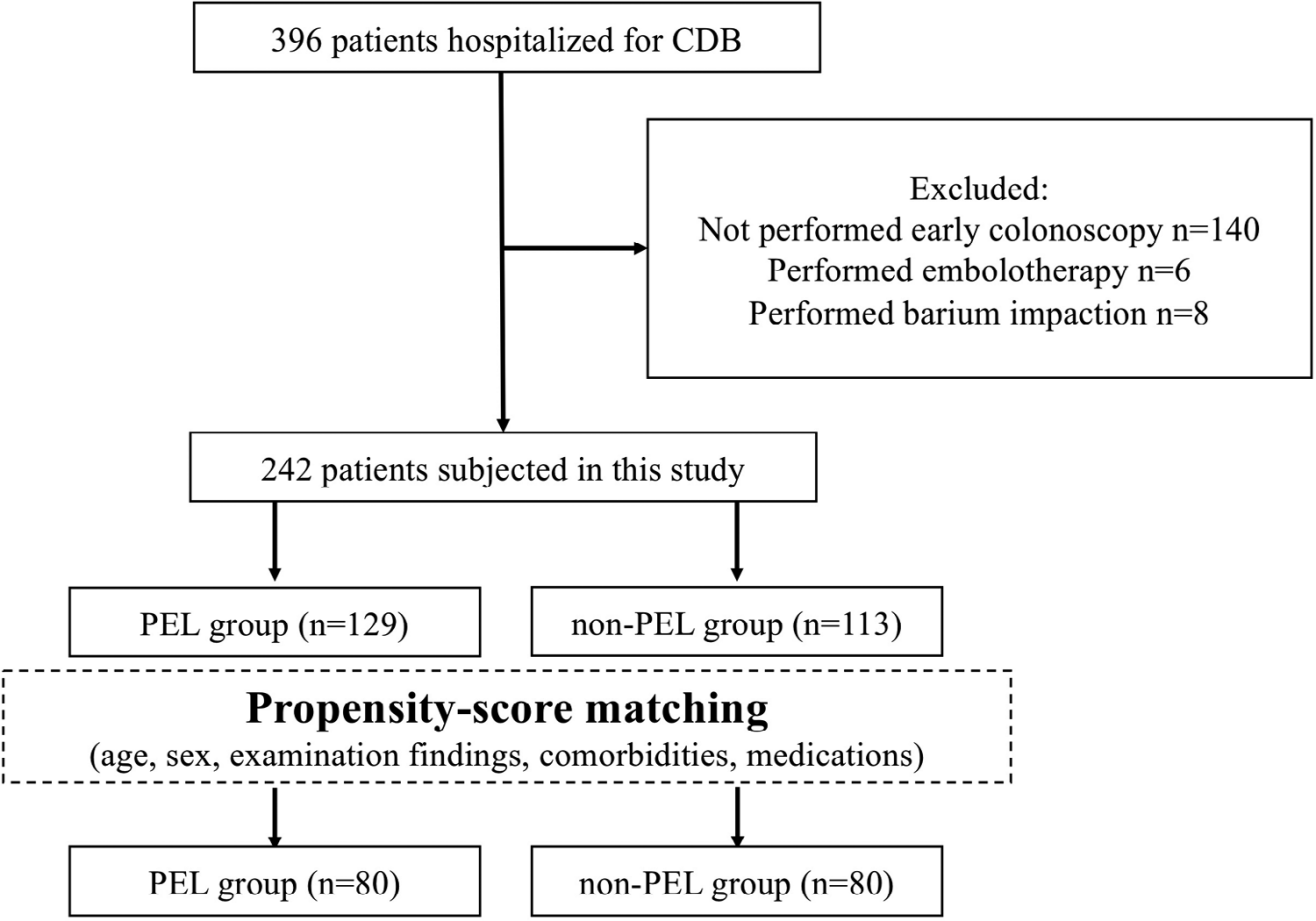
Study flowchart. CDB, colonic diverticular bleeding; PEL, polyethylene glycol lavage.

A total of 396 patients were hospitalized for CDB during the study period. Of these, 140 patients were excluded because they did not undergo early colonoscopy, and 14 were excluded because they underwent non‐endoscopic hemostatic therapy, including therapeutic embolization or barium impaction. The study inclusion flowchart is shown in Figure 1.

Table 1 shows the clinical and procedural characteristics of the 242 patients included in the study. The median age was 76 years, and 60.7% of patients were men. The median hospital stay was 8 days. On admission, the median systolic arterial pressure was 131 mm Hg, and the median hemoglobin level was 11.2 g/dL. Approximately half of the patients had taken antithrombotic agents, 15.7% had taken nonsteroidal anti‐inflammatory drugs, and 34.4% received a blood transfusion. Approximately half of the patients (*n* = 129) underwent bowel preparation with PEL prior to early colonoscopy. The cecal intubation rate was 70.3%, and the median observation time was 19 min. The SRH were observed in 35.5% of patients, and six patients had colonoscopy‐related adverse events; four patients had abdominal fullness, one experienced vomiting, and one experienced a vagal reflex. Endoscopic hemostasis was performed in 67 patients. A total of 21 patients (8.7%) experienced rebleeding within 1 week, 29 (12.0%) within 1 month, and 59 (24.0%) within 1 year. A single patient died of a cerebral infarction during hospitalization.

**TABLE 1:** Patient and procedural characteristics.

Variable	Value	Missing values
Age, year, median (range)	76 (39–102)	
Male sex, *n* (%)	147 (60.7)	
Hospitalization, days, median (range)	8 (1–33)	
SAP, mm Hg, median (range)	131 (77–210)	
Hgb at admission, g/dL, median (range)	11.2 (4.0–17.9)	
Comorbidities, *n* (%)		
Hypertension, *n* (%)	165 (68.2)	
Diabetes mellitus, *n* (%)	43 (17.8)	
Chronic kidney disease, *n* (%)	26 (10.7)	
Malignant neoplasm, *n* (%)	14 (5.8)	
Performance status		
0, *n* (%)	132 (54.5)	
1, *n* (%)	65 (26.9)	
2, *n* (%)	27 (11.2)	
3–4, *n* (%)	18 (7.4)	
Antithrombotic agents, *n* (%)	115 (47.5)	
Antiplatelet agent	58 (24.0)	
Anticoagulant agent	27 (11.2)	
Multiple agents	30 (12.4)	
NSAIDs, *n* (%)	38 (15.7)	
Blood transfusion, *n* (%)	83 (34.3)	
Colonoscopy		
Bowel preparation, *n* (%)	129 (53.3)	
Cecal intubation, *n* (%)	168 (70.3)	3 (1.2)
Observation time, min, median (range)	19 (1–121)	7 (2.9)
Adverse event, *n* (%)	6 (2.5)	
Abdominal pain	4 (1.7)	
Vomiting	1 (0.4)	
Vagal reflex	1 (0.4)	
SRH, *n* (%)	86 (35.5)	
Active bleeding	32 (13.2)	
Adherent clot	47 (19.4)	
Visible vessel	7 (2.9)	
Endoscopic treatment, *n* (%)	67 (27.7)	
Clipping	47 (19.4)	
Band ligation	20 (8.3)	
Rebleeding within 1 week, *n* (%)	21 (8.7)	
Rebleeding within 1 month, *n* (%)	29 (12.0)	
Rebleeding within 1 year, *n* (%)	58 (24.0)	

Abbreviations: Hgb, hemoglobin; NSAIDs, nonsteroidal anti‐inflammatory drugs; SAP, systolic arterial pressure; SRH, stigmata of recent hemorrhage.

Table 2 shows a comparison of clinical findings between PEL and non‐PEL groups. The PEL group was significantly younger (*p* = 0.036), had fewer patients with hypertension (*p* = 0.027) and malignant neoplasm (*p* = 0.002), and had better performance statuses (*p* = 0.018). Patients in the PEL group received fewer blood transfusions (*p* = 0.002), had a higher rate of cecal intubation (*p* < 0.001), and shorter observation times (*p* = 0.006). The length of hospital stay was shorter in the PEL group (*p* < 0.001). There were no significant differences in adverse event rates and the rates of SRH identification, endoscopic hemostatic treatment, and rebleeding within 1 week.

**TABLE 2:** Patient and procedural characteristics by group: polyethylene glycol lavage versus non‐polyethylene glycol lavage.

	PEL group (*n* = 129)	Missing values	Non‐PEL group (*n* = 113)	Missing values	SD*	*p*‐value
Age, year, mean ± SD	72.2 ± 12.1		75.4 ± 13.2		0.253	0.036
Male sex, *n* (%)	73 (56.6)		74 (65.5)		0.149	0.16
SAP, mm Hg, mean ± SD	130.1 ± 27.9		131.4 ± 25.5		0.049	0.70
Hgb at admission, mean ± SD	11.3 ± 2.5		10.7 ± 2.5		0.240	0.063
Hypertension, *n* (%)	80 (62.0)		85 (75.2)		0.230	0.027
Diabetes mellitus, *n* (%)	20 (15.5)		23 (20.4)		0.106	0.33
Chronic kidney disease, *n* (%)	10 (7.8)		16 (14.2)		0.176	0.11
Malignant neoplasm, *n* (%)	2 (1.6)		12 (10.6)		0.358	0.002
Performance status						
0, *n* (%)	79 (32.6)		53 (21.9)		0.194	
1, *n* (%)	32 (13.2)		33 (13.6)		0.010	0.018
2, *n* (%)	14 (5.8)		13 (5.4)		0.014	
3 or 4, *n* (%)	4 (1.7)		14 (5.8)		0.195
Antithrombotic agents, *n* (%)						
Antiplatelet agent	32 (24.8)		26 (23.0)		0.034	
Anticoagulant agent	15 (11.6)		12 (10.6)		0.026	0.88
Multiple agents	14 (10.9)		16 (14.2)		0.083	
None	68 (52.7)		59 (52.2)		0.008	
NSAIDs, *n* (%)	19 (14.7)		19 (16.8)		0.048	0.66
Blood transfusion, *n* (%)	33 (25.6)		50 (44.3)		0.334	0.002
Colonoscopy						
Cecal intubation, *n* (%)	113 (88.3)	1	55 (49.6)	2	0.810	< 0.001
Observation time, min, mean ± SD	20.8 ± 16.2	5	29.9 ± 24.8	2	0.434	0.006
Adverse event, *n* (%)	2 (1.6)		4 (3.5)		0.105	0.32
SRH, *n* (%)	46 (35.7)		40 (35.4)		0.005	0.97
Endoscopic treatment, *n* (%)	40 (31.0)		27 (23.9)		0.129	0.22
Rebleeding within 1 week, *n* (%)	12 (9.3)		9 (8.0)		0.037	0.71
Hospitalization, days, mean ± SD	8.0 ± 5.1		10.9 ± 5.8		0.531	< 0.001

Abbreviations: Hgb, hemoglobin; NSAIDs, nonsteroidal anti‐inflammatory drugs; PEL, polyethylene glycol lavage; SAP, systolic arterial pressure; SD*, standardized difference; SD, standard deviation; SRH, stigmata of recent hemorrhage.

Table 3 shows the results after propensity score matching, which included 80 patients in each group. There were no significant differences in the rate of blood transfusion and observation time, rates of adverse events, identification of SRH, endoscopic hemostatic treatment, or rebleeding within 1 week. However, even after matching, the PEL group had a higher rate of cecal intubation (*p* < 0.001) and significantly shorter hospital stays (*p* = 0.001).

**TABLE 3:** Patient and procedural characteristics after propensity score matching by group: polyethylene glycol lavage versus non‐polyethylene glycol lavage.

	PEL group (*n* = 80)	Missing values	Non‐PEL group (*n* = 80)	Missing values	SD*	*p*‐value
Age, year, mean ± SD	73.5 ± 10.5		74.3 ± 12.5		0.069	0.55
Male sex, *n* (%)	51 (63.8)		50 (62.5)		0.022	0.87
SAP, mm Hg, mean ± SD	133.4 ± 28.9		130.2 ± 24.8		0.119	0.45
Hgb at admission, g/dL, mean ± SD	11.4 ± 2.4		11.0 ± 2.6		0.160	0.33
Hypertension, *n* (%)	58 (72.5)		59 (73.8)		0.024	0.86
Diabetes mellitus, *n* (%)	15 (18.8)		17 (21.3)		0.051	0.69
Chronic kidney disease, *n* (%)	10 (12.5)		9 (11.3)		0.030	0.81
Malignant neoplasm, *n* (%)	2 (2.5)		2 (2.5)		0.000	1.00
Performance status						
0, *n* (%)	45 (56.3)		43 (53.8)		0.041	
1, *n* (%)	24 (30.0)		26 (32.5)		0.044	0.79
2, *n* (%)	7 (8.8)		9 (11.3)		0.069	
3 or 4, *n* (%)	4 (5.0)		2 (2.5)		0.102	
Antithrombotic agents, *n* (%)						
Antiplatelet agent	20 (25.0)		20 (25.0)		0.000	
Anticoagulant agent	9 (11.3)		8 (10.0)		0.034	0.98
Multiple agents	10 (12.5)		9 (11.3)		0.030	
None	41 (51.3)		43 (53.8)		0.041	
NSAIDs, *n* (%)	12 (15.0)		14 (17.5)		0.056	0.67
Blood transfusion, *n* (%)	22 (27.5)		32 (40.0)		0.221	0.094
Colonoscopy						
Cecal intubation, *n* (%)	72 (91.1)	1	39 (50.0)	2	0.905	<0.001
Observation time, min, mean ± SD	20.2 ± 13.6	2	29.4 ± 25.0	2	0.457	0.058
Adverse event, *n* (%)	1 (1.3)		3 (3.8)		0.142	0.30
SRH, *n* (%)	29 (36.5)		28 (35.0)		0.026	0.87
Endoscopic treatment, *n* (%)	24 (30.0)		22 (27.5)		0.045	0.73
Rebleeding within 1 week, *n* (%)	8 (10.0)		7 (8.8)		0.033	0.79
Hospitalization, days, mean ± SD	7.9 ± 4.7		10.1 ± 5.2		0.444	0.001

Abbreviations: Hgb, hemoglobin; NSAIDs, nonsteroidal anti‐inflammatory drugs; PEL, polyethylene glycol lavage; SAP, systolic arterial pressure; SD*, standardized difference; SD, standard deviation; SRH, stigmata of recent hemorrhage.

## DISCUSSION

In this multicenter retrospective study, we used propensity score matching to clarify the benefits of PEL for early colonoscopy in patients with CDB. To our knowledge, this is the first study to evaluate the efficacy of PEL for early colonoscopy in patients with CDB using propensity score matching analysis.

In a previous study, 66.4% of patients with ALGIB who underwent early colonoscopy were prepared with PEL.^1^ In our study of patients with CDB, 53.3% were prepared with PEL. These results suggest that there is no standard for bowel preparation in early colonoscopy and that the decision depends on the attending physician. Both the Japanese guidelines for CDB and international guidelines for patients with ALGIB recommend bowel preparation with PEL.^13^, ^15^, ^16^ However, these recommendations are not supported by sufficient evidence. Several studies have reported that PEL provides high diagnostic and cecal intubation rates in patients with ALGIB,^17^, ^18^ but these studies did not adjust for patient's background characteristics. In the present study, after propensity score matching was used to reduce selection bias, the PEL group had shorter hospital stays and higher cecal intubation rates than the non‐PEL group. These results suggest that bowel preparation with PEL prior to early colonoscopy for ALGIB may benefit the subsequent clinical course.

Reportedly, CDB occurs more frequently in the right colon than in the left colon.^9^ This suggests that cecal intubation may influence the diagnosis and treatment of CDB. In addition, PEL preparation allows for the easy confirmation of a hemostatic state after colonoscopy because the lavage effect confirms nonhematologic discharge. In patients who do not undergo PEL, the presence of residual bloody stools can make it difficult to determine whether bleeding is continuing after colonoscopy. This presumably contributed to the delayed time of hemostasis confirmation observed in our non‐PEL group, which in turn presumably resulted in a longer hospital stay.

Adequate bowel preparation with PEL requires at least 3–4 h.^14^, ^19^ Some reports have suggested that this additional time can be a barrier to immediate colonoscopy in patients with severe ALGIB.^20^ However, we did not observe any significant differences in the rate of blood transfusion, rebleeding, or adverse events in the PEL group compared with the non‐PEL group. One patient in the non‐PEL group died of a cerebral infarction in this study, although this event was not associated with PEL. Early colonoscopy with bowel preparation in patients with ALGIB reportedly has a low adverse event rate, similar to that of colonoscopy in patients who are not experiencing gastrointestinal bleeding.^21^ Therefore, we believe that the additional time required for PEL will not have a negative effect on the clinical outcome of patients with CDB, and that early colonoscopy with PEL in patients with CDB has comparable safety to the procedure performed without PEL. On the other hand, CDB is reported to be more common in the elderly patients.^1^, ^22^ It has been reported that elderly patients, especially those with poor performance status who have difficulty with ambulation, are at the risk of inadequate bowel preparation even while undergoing PEL.^23^ Therefore, it may be necessary to consider whether preparation with PEL should be performed in elderly patients, taking into account their general condition.

This study has several limitations. First, the retrospective nature introduces selection bias that cannot be completely excluded even with the use of propensity score matching analysis. Second, although this is a multicenter study, the sample size was still small to verify the clinical efficacy of bowel preparation for early colonoscopy in patients with CDB. Finally, clinical decisions, including hospital discharge, were not strictly standardized, and detailed clinical information, such as the type of colonoscope, the presence or absence of sedation, and the level of endoscopist, was not collected due to the retrospective multicenter study design. A prospective multicenter cohort study is needed to validate our results.

This study shows that bowel preparation using PEL results in shorter hospital stays and higher cecal intubation rates in early colonoscopy for patients with CDB. In addition, the safety of early colonoscopy with PEL is comparable to the safety of colonoscopy without PEL. These results suggest that early colonoscopy for patients with CDB should be performed with PEL whenever possible. Prospective multicenter studies are needed to confirm our results.

## CONFLICT OF INTEREST STATEMENT

Authors declare no conflicts of interest for this article. Author Takayuki Matsumoto is an advisory member of DEN Open.
